# RUNX2-modifying enzymes: therapeutic targets for bone diseases

**DOI:** 10.1038/s12276-020-0471-4

**Published:** 2020-08-13

**Authors:** Woo-Jin Kim, Hye-Lim Shin, Bong-Soo Kim, Hyun-Jung Kim, Hyun-Mo Ryoo

**Affiliations:** Basic Research Lab for “Epigenetic Regeneration of Aged Skeleto-Muscular System (ERASMUS)”, Department of Molecular Genetics and Dental Pharmacology, School of Dentistry, Dental Research Institute, Seoul National University, Seoul, South Korea

**Keywords:** Osteoporosis, Acetylation

## Abstract

RUNX2 is a master transcription factor of osteoblast differentiation. RUNX2 expression in the bone and osteogenic front of a suture is crucial for cranial suture closure and membranous bone morphogenesis. In this manner, the regulation of RUNX2 is precisely controlled by multiple posttranslational modifications (PTMs) mediated by the stepwise recruitment of multiple enzymes. Genetic defects in *RUNX2* itself or in its PTM regulatory pathways result in craniofacial malformations. Haploinsufficiency in RUNX2 causes cleidocranial dysplasia (CCD), which is characterized by open fontanelle and hypoplastic clavicles. In contrast, gain-of-function mutations in FGFRs, which are known upstream stimulating signals of RUNX2 activity, cause craniosynostosis (CS) characterized by premature suture obliteration. The identification of these PTM cascades could suggest suitable drug targets for RUNX2 regulation. In this review, we will focus on the mechanism of RUNX2 regulation mediated by PTMs, such as phosphorylation, prolyl isomerization, acetylation, and ubiquitination, and we will summarize the therapeutics associated with each PTM enzyme for the treatment of congenital cranial suture anomalies.

## Regenerative medicine: Manipulating modifications that control bone-building

Therapies that modulate the activity of the regulatory protein RUNX2 could potentially restore normal bone development in a range of skeletal disorders, and repair damage from injury or degeneration. RUNX2 is an essential regulator of genes that drive formation of bone-producing osteoblast cells. It can be activated or inactivated by the enzymatic addition of various chemical groups. Hyun-Mo Ryoo and colleagues at Seoul National University, South Korea, review the role of such modifications in bone disorders. For example, the loss of modifications activated by RUNX2 can result in delayed integration of the bones that form the skull. The authors highlight potential opportunities to manipulate these modification processes to treat this and other developmental disorders. Similar strategies could also promote repair of fractures or counter osteoporotic bone loss.

## Introduction

As indicated in the “Central Dogma of Molecular Biology”, the information encoded in DNA is replicated for cell doubling, and this information is transcribed into RNA and translated into protein for maintaining the life of the cell^1^. Genetic diseases are caused by abnormal information in parental DNA that is inherited by descendants. The development of the CRISPR-Cas9 system creates the possibility of genetic correction of misinformation in the zygote stage to overcome genetic diseases^2^. Additionally, recent advances in high-throughput genomic sequencing have dramatically enhanced the accumulation of knowledge on the genetic aspects of diseases^3^. Unfortunately, we understand that there are still many obstacles, such as technical, ethical, and legal problems, involved in making this vision come true^4^. In the case of genetic diseases caused by single-gene defects, we can design an alternative way to alleviate genetic defects with our increased understanding of gene expression and protein regulation^5^. Since the function of RUNX2 was first identified in 1997^6^, profound knowledge about the gene expression regulation, protein–protein interactions, and posttranslational modifications (PTMs) of RUNX2 has accumulated. Here, we reviewed therapeutic approaches associated with regulatory mechanisms of RUNX2 mediated by PTMs to address anomalies in congenital craniofacial suture morphogenesis.

## RUNX2 expression in cranial suture closure and membranous bone morphogenesis

RUNX2 is expressed in membranous bones and endochondral bones^7^. In situ hybridization has indicated that *Runx2* mRNA expression occurs in undifferentiated sutural mesenchymal cells, committed preosteoblasts in osteogenic fronts and developing membranous parietal bones in the early stage at embryonic day 15.5 (E15.5)^8^. The osteogenic signal in the membranous bones becomes weaker and weaker with the maturation of the membranous bones at E17.5 and E18.5, indicating that *Runx2* expression fades as osteoblasts and osteocytes mature^9^. The *Runx2-II* isoform is bone specific, and its expression is found only in osteogenic fronts and bone-forming osteoblasts. On the other hand, the *Runx2-I* isoform is expressed in sutural mesenchymal cells in addition to the *Runx2-II* expression area^8,9^. These results clearly indicate the importance of RUNX2 in cranial suture morphogenesis. In this sense, delayed cranial suture closure phenotypes are expected in cleidocranial dysplasia (CCD) patients in which one allele of *RUNX2* has loss-of-function mutations. Recently, many *RUNX2* mutations have been identified in CCD patients. The mutation sites are widely distributed throughout the functional domains of RUNX2^10^. In contrast, the major causative factors in craniosynostosis (CS), which is characterized by early suture closure and associated skull malformation and craniofacial deformity, are closely associated with the RUNX2 regulation mechanism^11,12^.

**Fig. 1:**
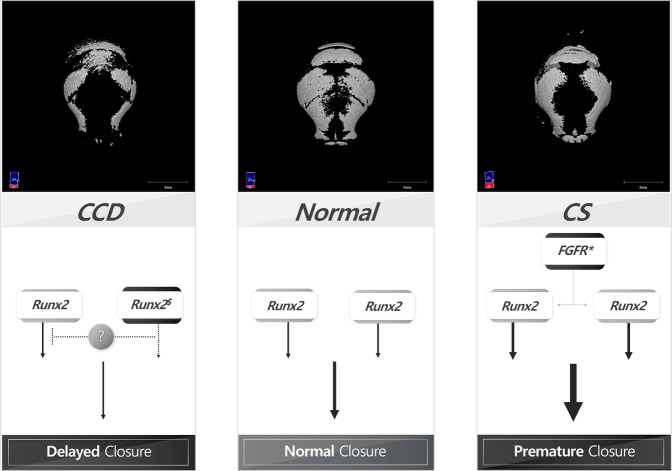
Hypothesis of the molecular mechanism involving RUNX2 in cranial suture anomalies. RUNX2 transcriptional, translational and PTM level regulations are closely associated with cranial suture anomalies. Based on the amount of RUNX2 expressed in the normal skull, hypoostotic disease, typically CCD, results from a reduced RUNX2 level in the cranial sutures due to haplodeficiency. In contrast, osteopenic disease, typically CS, results from an increased RUNX2 level in the cranial sutures due to constitutive FGFR2 signaling. (asterisk means hyper-active mutation of gene; $ means hypo-active mutation of gene).

Many signals, such as FGF, BMP, and Shh, are involved in cranial suture closure^13^. FGF signaling^12^ and BMP signaling^14^ regulate RUNX2 expression, and RUNX2 regulates FGF2-induced BMP2 expression^15^ and Shh signaling^16^. These results indicate that RUNX2 must be a central regulator of cranial suture closure and that it mutually regulates and is regulated by these signaling molecules. Therefore, RUNX2 or RUNX2-associated signaling pathways could be therapeutic targets in cranial suture anomalies (Fig. 1).

## Regulation of RUNX2 PTMs

Many osteoinductive signaling pathways participate in cranial development; among these pathways, the pathways involving FGFs and their cognate receptors, FGFRs, are important signaling pathways involved in cranial suture morphogenesis. FGF2, a basic FGF, is a strong mitogen for bone-derived cells^17^. Disruption of the *Fgf2* gene results in decreased bone formation^18^. Constitutively active mutations of *Fgfr*1, *Fgfr*2, and *Fgfr*3 cause CS. In osteoblasts, the ligand binding of FGFs such as FGF2 with the corresponding FGFRs induces receptor dimerization and autophosphorylation of receptor tyrosine kinases, which in turn activates multiple signal transduction pathways, including those involving mitogen-activated protein kinases (MAPKs) and protein kinase C (PKC)^11,12^. We were the first to identify that FGF-FGFR signaling enhances RUNX2 mRNA expression and posttranslational protein activation^12^. PKC plays a central role in the stimulation of RUNX2 gene expression^12^. On the other hand, ERK or p38 MAP kinases are involved in RUNX2 protein activation but do not affect RUNX2 mRNA expression^12^.

As indicated in many reviews, RUNX2 is precisely regulated by phosphorylation, and many phosphorylation sites exist in this molecule^19,20^. Several kinases can phosphorylate discrete ranges of target amino acid residues in RUNX2. For example, PKC, which is activated by FGF2, phosphorylates Ser247 in the RUNX2 protein^11^. ERK, which is activated by FGF signaling, phosphorylates the Ser301 residue in the PST domain, which is critical for the enhancement of subsequent acetylation and the suppression of ubiquitination of the RUNX2 protein^21^. Moreover, in the BMP2-induced RUNX2 activation process, RUNX2 acetylation^22^ by ERK-activated p300^23^, an acetyl transferase, is critical. These results indicate that RUNX2 phosphorylation and other RUNX2 modifications have linear correlations with subsequent RUNX2 acetylation. The acetylation of lysine residues in RUNX2 protects the protein from ubiquitination, which consequently stabilizes and activates RUNX2 transactivation activity^21^. In the connection between RUNX2 phosphorylation and acetylation, RUNX2 phosphorylation alone is not sufficient to explain remote lysine residue acetylation.

A peptidyl prolyl *cis–trans* isomerase, PIN1, makes a pivotal new twist in peptide bonds between phosphorylated serine or threonine and proline. This *cis–trans* isomerization creates a tremendous structural shift on the other side of the peptide bond^24^. A previous review indicated that PIN1 is mainly localized in the nucleus and modifies nuclear transcription factors as well as various nuclear enzymes^25^. More recently, PIN1 activity has also been shown to modify downstream mediators of FGF, BMP, and WNT signaling^26^. Yoon et al. indicated that without *cis–trans* isomerization, phosphorylated proteins cannot be acetylated but can be ubiquitinated^24^. In addition, some lysine residues are not acetylated until the pSer/pThr-Pro peptide bond is twisted by PIN1^24,27^. Collectively, the RUNX2 protein phosphorylated in response to FGF signaling undergoes immediate isomerization by PIN1, and the consequent *cis–trans* structural modification exposes lysine residues that become acetylated. These serial PTM steps are reviewed in Kim et al. ^28^. Although we can control RUNX2 expression via stimulation with several extracellular ligands, direct control of RUNX2 activity is relatively complicated. As RUNX2 undergoes these serial PTMs and RUNX2 transactivation activity is regulated by these modifications, we can regulate RUNX2 activity by modulating these enzymatic activities with specific inhibitors of these enzymes. As summarized in previous studies^28^, RUNX2 activation can be achieved only by HDAC inhibitors (HDIs) or direct protein augmentation, but RUNX2 inhibition can be induced by all other inhibitors of PTM enzymes.

## Treatment of CS by regulation of RUNX2 posttranslational modification

CS which is defined as the premature fusion of one or more of the cranial sutures, presents as secondary distortion of the skull^29^ and midfacial hypoplastic changes^30^ that occur because of the combination of a lack of growth perpendicular to the fused sutures, compensatory overgrowth at the nonfused sutures, and structural interrelation between cranial bony components^31^. Most genetically determined CS is characterized by autosomal dominant inheritance, and the genes most commonly mutated in CS are *FGFR2*, *FGFR3*, *TWIST1*, and *EFNB1*^32^. Among these mutations, the Ser252Trp and Pro253Arg missense mutations in *FGFR2* cause Apert syndrome, which is characterized by bicoronal synostosis, symmetrical syndactyly, and complex craniofacial deformities, including midfacial hypoplasia and cleft palate^33^, and *Fgfr2*^*S252W*^, and *Fgfr2*^*P253R*^ mutations are well established in mouse models^34^.

The identification of pathologically activated FGF-signaling molecules in CS raises the possibility of screening candidates for pharmacological therapy. The FGF-MAPK and FGF-PKC signaling axes directly regulate Runx2 activation and expression and could be primary candidates for CS treatment^12,35^. The receptor tyrosine kinase domain of FGFR activates several MAP kinases, such as ERK and p38, and chemical inhibition of these MAP kinase-signaling pathways rescues abnormal skeletal phenotypes in models with *Fgfr2*^*S252W*^ or *Fgfr2*^*P253R*^ substitution mutations^36^.

The posttranslational regulation of RUNX2 by subsequent phosphorylation and acetylation is essential for FGF-induced cranial development, and these events could also be strong candidate strategies. Knocking out *Pin1*, an important RUNX2 regulatory isomerase, in mice severely suppresses osteogenesis in the developmental stage in vitro and in vivo^37^ and weakens the subnuclear focal accumulation of RUNX2^24^. In this manner, the genetic or pharmacological inhibition of PIN1 attenuates phenotypes in an Apert syndrome mouse model^29^. In a study with a genetic approach, crossbreeding *Fgfr2*^*S252W*^-heterozygous and *Pin1*-heterozygous mice significantly rescued the premature cranial suture closure and abnormal cranial shape^29^ and midface hypoplasia^30^ seen in *Fgfr2*^*S252W*^ mutant mice. Pharmacological suppression of PIN1 activity also effectively rescues CS phenotypes in *Fgfr2*^*S252W*^ mutant mice. These results highlight that the inhibition of PIN1 represents a viable alternative to surgical intervention for the treatment of CS and other hyperostotic diseases.

**Fig. 2:**
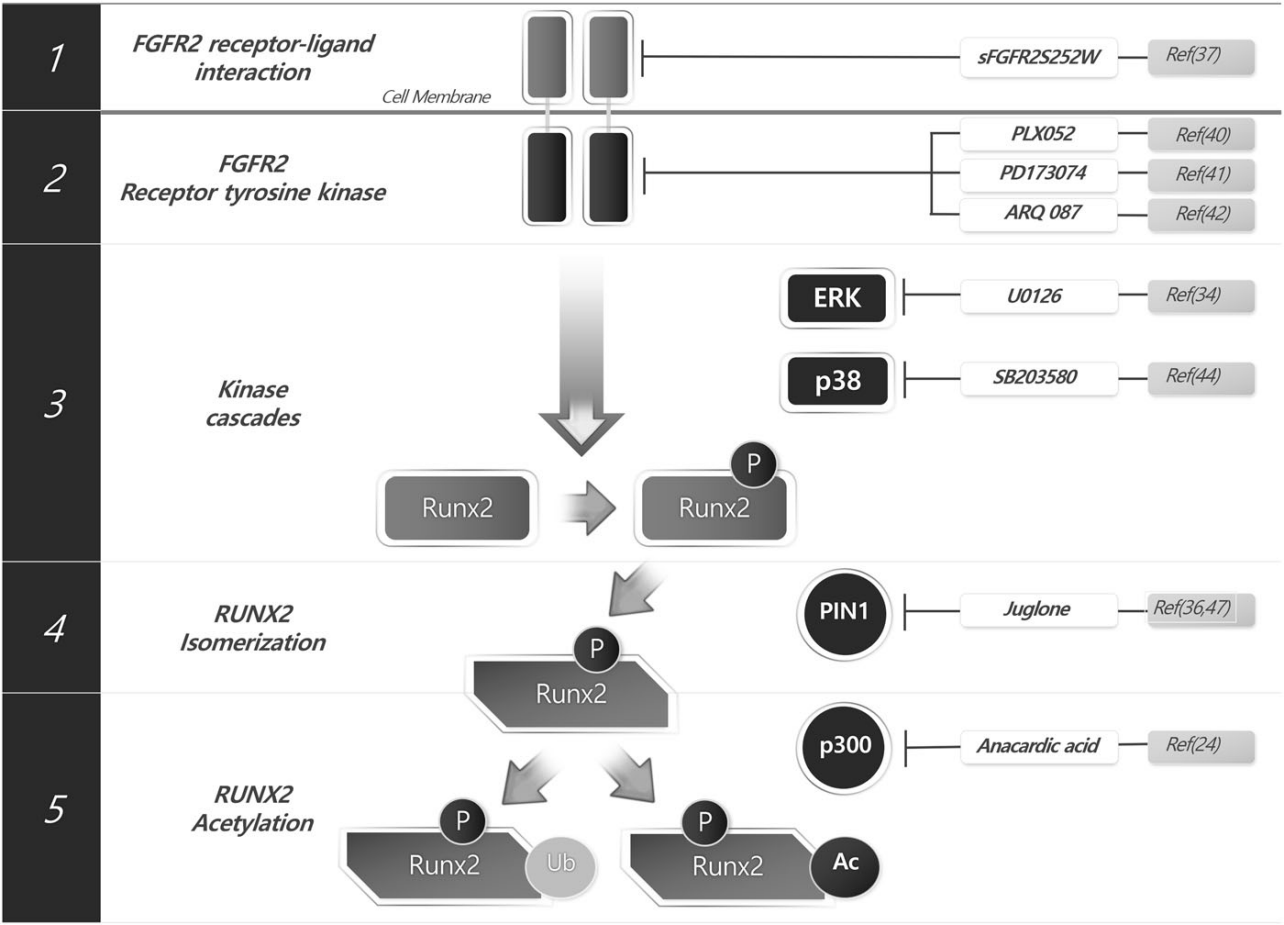
Therapeutic intervention points targeting RUNX2 activity regulation for the treatment of craniosynostosis. The soluble FGFR2 decoy receptor sFGFR2S252W may sequester and inhibit FGF ligands from binding to cell surface receptors. The receptor tyrosine kinase inhibitors PLX052, PD173074 and ARQ087 may block ligand binding-induced signaling propagation to cytosolic mediators. The MEK1/2 inhibitor U0126 or p38 inhibitor SB203580 blocks cytosolic mediator or nuclear transcription factor phosphorylation. The PIN1 inhibitors juglone and DTM block cis-trans structural modification of phosphorylated proteins. The HAT inhibitor anacardic acid blocks protein stabilization and activation by acetylation.

The accumulation of new findings regarding the FGF-Runx2 regulatory mechanism in cranial sutures opens the potential for additional therapeutic options for CS treatment. As shown in Fig. 2, FGF signaling can be summarized in five steps. First, FGF signaling is activated by a ligand–receptor interaction that results in the autophosphorylation of tyrosine residues in the intracellular region of FGFR. Second, the receptor tyrosine kinase signal is relayed through the phosphoinositide phospholipase C (PLC)-PKC and ERK pathways. Third, ERK-mediated and PKC-mediated phosphorylation occurred in RUNX2. Fourth, phosphorylated RUNX2 is immediately isomerized by PIN1, exposing the acetylation site. Fifth, the exposed lysine residues are acetylated by a histone acetyl transferase (HAT), increasing the stability of RUNX2. Consequently, by inhibiting the above steps with specific inhibitors, we can regulate the pathological activation of RUNX2 for CS treatment (Fig. 2).

In the ligand–receptor interaction step, a soluble decoy receptor or ligand–receptor interaction inhibitor could be candidates. In a recent study, nanogel-mediated delivery of a purified soluble form of FGFR2 carrying the S252W mutation (sFGFR2^S252W^) was tested in calvarial tissue cultures from Apert syndrome model mice^38^. Additionally, Mooney et al. reported that the application of an anti-TGF-β2 antibody inhibited postoperative resynostosis in a rabbit CS model^39^.

Although originally developed as anticancer agents, multiple FGFR kinase inhibitors are also currently being investigated as potential treatment options for CS^40^. The FGFR tyrosine kinase inhibitors PLX052^41^ and PD173074^42^ have been reported to successfully prevent premature suture fusion in a model with the C342Y mutation in FGFR2. Another FGFR tyrosine kinase inhibitor, ARQ-087^43^, has also been shown to suppress the hyperactivation of mutant FGFR1, FGFR2, and FGFR3 signaling.

After receptor kinase activation, downstream signaling molecules are also good targets for treatment. The MEK1 inhibitor PD98059 was tested in calvarial tissue obtained from an Apert syndrome (*Fgfr*^*P253R*^) mouse model and produced partial alleviation of premature coronal suture fusion^44^. In another study, the MEK1/2 inhibitor U0126 was injected intraperitoneally into pregnant mice with the *Fgfr2*^*S252W*^ mutation, and the offspring were phenotypically normal at birth, including the patency of the coronal sutures^36^. A p38 kinase inhibitor (SB203580) has been employed to treat *Fgfr2*^*Y394C*^ mice (an animal model of Beare–Stevenson cutis gyrata syndrome), which are characterized by CS and epidermal hyperplasia^45^. Although the mice that received injections of SB203580 in utero had significantly ameliorated the incidence of skin abnormalities, there was no obvious improvement in the skull phenotype and neonatal lethality in *Fgfr2*^*+/Y394C*^ mice^45^. These results indicate that MEK1/ERK signaling is more closely related to the pathogenesis of FGFR2 mutation-related CS than p38 signaling.

The phospho-serine/threonine and proline sequences of RUNX2 become the substrate of the isomerase PIN1. The *cis–trans* isomerization mediated by PIN1 can be blocked by well-known inhibitors such as juglone^46^ and DTM^47^. Intraperitoneal administration of juglone to pregnant *Fgfr2*^*S252W*^ mice from E14.5 to E18.5 was found to successfully interrupt fetal development of Apert syndrome phenotypes, including early suture closure^29^ and midfacial deformalities^30^. Additionally, the expression levels of the FGFR2 downstream genes Dusp6, Spry2, and Spry3 are attenuated by juglone treatment in primary cultured osteoblasts from *Fgfr2*^*S252W*^ mice^29,30^. Posttranslational stabilization and activation of RUNX2 in *Fgfr2*^*S252W*^ osteoblasts are also attenuated by PIN1 inhibition^29^.

We previously suggested that the isomerization of RUNX2 subsequently induced acetylation by p300^21^. Treatment with the p300 inhibitor anacardic acid^48^ dramatically suppresses FGF2-stimulated Runx2 activity, whereas the administration of trichostatin A (HDI) synergistically augments Runx2 activity^24^. These results do not directly define the HAT inhibitor as a CS therapeutic, but Runx2 activation by FGF2 is crucially associated with lysine acetylation by p300, and the histone deacetylase inhibitor represents a viable alternative for CS treatment.

## Treatment of CCD by inhibition of RUNX2 deacetylation

As RUNX2 is an indispensable transcription factor of osteogenesis and osteoblast differentiation and heterozygotic RUNX2 loss-of-function mutation causes delayed cranial suture closure, it is natural to consider RUNX2-activating agents to be candidate therapeutics for CCD as well as hypoostotic conditions such as osteoporosis. Despite extensive research and drug development of protein inhibitors, the development of drugs for protein activation is limited^49^. Because of the natural moiety of small chemical drugs, these drugs are effective in inhibiting actions mediated through structural changes in a target protein site but are not suitable for activating the target protein^50^. To overcome this limitation, many alternatives to target activation have been explored, such as gene therapy, protein augmentation, or bypassing the inhibition of target-inhibiting pathways.

Naturally, signaling cascades are terminated by protein ubiquitination, and the rate of ubiquitination is an important factor in the maintenance and intensity of target signaling^51^. As described above, many osteogenic signaling pathways ultimately result in RUNX2 activation, and this activity is precisely regulated by phosphorylation and isomerization, which is critical for the enhancement of the subsequent acetylation and suppression of the ubiquitination of the RUNX2 protein^21^. In this manner, a strategy for increasing the level of RUNX2 protein acetylation is a unique alternative to compensating for a reduced RUNX2 level. Many HDIs, such as SAHA^52^, TSA^53^, valproic acid^54^, sodium butyrate^55^, and MS-275^56^, have been indicated as therapeutics for osteopenic conditions. Among these HDIs, MS-275 has shown strong osteogenic effects in cell culture and animal studies^56^.

MS-275 has been studied as a CCD treatment^57^. Because HDIs have strong RUNX2-enhancing activities, they were applied to pregnant *Runx2*^*+/*−^ mice^57^. As cranial bone formation begins at E14.5^6^, MS-275 was intraperitoneally injected beginning on E14.5. When it was injected only once, its osteogenic effect was apparent at E16.5 but had disappeared at birth, which indicates that pharmacokinetically, MS-275 activity was not sustained 4–5 days after injection. Double injection at E14.5 and E16.5 was sufficient to show a therapeutic effect at birth. The clavicle is one of the earliest forming bones (around E13.5)^58^ in mice and humans, so E14.5 injection appears to be a little late to recover clavicle development. Both alleles of *Runx2* contribute to the expressed RUNX2 level for bone development, so a loss-of-function mutation in one allele causes *Runx2* haplodeficiency. MS-275 treatment opens a novel therapeutic avenue to treat CCD by compensating for genetic RUNX2 insufficiency by metabolic stabilization. In addition, MS-275 may influence RUNX2 transcription by modifying histones around the RUNX2 promoter or enhancers^57^. Unfortunately, it is very difficult to determine which effect is the major mechanism of action of MS-275 in CCD therapy.

Although many HDIs show osteogenic effects that increase calvarial bone density, they do not effectively enhance cranial suture closure. In the case of SAHA, TSA, or valproic acid treatment enhanced additional new bone mineralization over original calvarial bones in a fluorescence-labeling experiment and micro-CT bone density analysis. In contrast to the above-mentioned HDIs, which share a hydroxamate functional group, MS-275 has a benzamide functional group. In terms of molecular targets, MS-275 has a specificity for Class I-HDACs, while the others have pan-HDAC or class II HDAC specificities^59^. This difference may explain why MS-275 is more effective in CCD treatment. We also do not exclude the possibility of a sufficient effect with MS-275 through the pharmacokinetic fine-tuning of other HDIs. Adjustments in the application dosage and treatment interval of other HDIs may also improve their therapeutic activity for CCD treatment.

Recently, some studies have attempted to solve protein dysfunction due to genetic problems through intracellular delivery of recombinant proteins^60^. The isomerization mediated by PIN1 consequently increases the activity and stability of RUNX2, so the recovery of the intracellular level of PIN1 is one of the solutions for osteopenic conditions. However, direct delivery of biomacromolecules, such as proteins, into the cytoplasm is quite challenging, and for this purpose, it is necessary to develop a carrier with high delivery efficiency and biocompatibility^61^. In our previous study, we reported an intracellular protein delivery system based on silk fibroin nanoparticle-encapsulated cationic lipid (DOTAP:DOPE) complexes^62^. Using this complex, we delivered recombinant PIN1 into *Pin1* knockout primary embryonic fibroblasts to attenuate the reduction in osteogenic differentiation in a *Pin1*-deficient model. Direct delivery of PIN1 led to increased ERK and SMAD signaling and resulted in recovery of osteogenic marker gene expression and mineral deposition in *Pin1*-deficient cells^63^. These preliminary results for the protein delivery application indicated that recombinant PIN1 delivery could be a potential therapeutic option for osteopenic conditions.

## Conclusion

Over the past 20 years, molecular and genetic advances have provided a new approach to dissecting the mechanisms underlying genetic bone diseases; success has mostly come from the identification of pathogenic signaling mechanisms, and recent advances in mechanism-based drug selection have dramatically enhanced the possibility of utilizing pharmacological approaches to treat genetic bone diseases. Enhanced RUNX2 acetylation by HDIs^57^ and direct PIN1 delivery^63^ may upregulate RUNX2 activity to rescue genetic *RUNX2* deficiency in CCD. FGFR2 receptor tyrosine kinase inhibitors^41–43^, downstream ERK inhibitors^36^ or PIN1 inhibitors^29,30^ can rescue FGFR2-hyperactivating mutations in CS. Previous results also suggest that a genetic disease caused by a single gene mutation can be overcome by regulation of the molecular metabolism associated with the causative protein. The understanding of the stepwise regulatory mechanism that controls RUNX2 PTMs may also be utilized for the treatment of fractures, bone regeneration, and hyperostotic or hypoostotic conditions. *RUNX2* repression is strongly associated with postmenopausal osteoporosis and breast cancer^64^. Additionally, enhanced expression of *RUNX2* is observed during fracture healing^65^, and *Runx2* is required for the early stages of endochondral bone formation^66^. In particular, MS-275 exerts a strong bone anabolic effect in models of osteoporosis, showing potential applications for treating bone disease via regulation of posttranslational modification of RUNX2^56^. Collectively, the enhanced stability of RUNX2 by posttranslational regulation could offer important candidates for bone regeneration therapy. Therapeutic candidates selected in these genetic bone diseases are also applicable to other diseases with the same regulatory mechanism. Proline phosphorylation and isomerization cascades are quite universal in various diseases, such as cancer^67^, Alzheimer’s disease^68^, vascular diseases^69^, allergic airway response^70^, and even infections^71^. Further research focused on identifying the disease-related roles of RUNX2 PTMs will broaden the horizon of drug candidates for genetic bone diseases and other diseases that share similar regulatory mechanisms. Overall, we can say that a broad and profound understanding of the molecular biology of a single causative gene in a congenital disease may provide us with a novel therapeutic avenue for genetic diseases as well as molecule-related diseases.

## References

[1] Crick F (1970). Central dogma of molecular biology. Nature.

[2] Knott GJ, Doudna JA (2018). CRISPR-Cas guides the future of genetic engineering. Science.

[3] Choy KW (2019). Next-generation sequencing to diagnose suspected genetic disorders. N. Engl. J. Med..

[4] Plaza Reyes A, Lanner F (2017). Towards a CRISPR view of early human development: applications, limitations and ethical concerns of genome editing in human embryos. Development.

[5] Smith M, Flodman PL (2018). Expanded insights into mechanisms of gene expression and disease related disruptions. Front. Mol. Biosci..

[6] Komori T (1997). Targeted disruption of Cbfa1 results in a complete lack of bone formation owing to maturational arrest of osteoblasts. Cell.

[7] Lee MH (2005). Dlx5 specifically regulates Runx2 type II expression by binding to homeodomain-response elements in the Runx2 distal promoter. J. Biol. Chem..

[8] Choi KY (2002). Spatio-temporal expression patterns of Runx2 isoforms in early skeletogenesis. Exp. Mol. Med..

[9] Park MH (2001). Differential expression patterns of Runx2 isoforms in cranial suture morphogenesis. J. Bone Min. Res..

[10] Ryoo HM, Kang HY, Lee SK, Lee KE, Kim JW (2010). RUNX2 mutations in cleidocranial dysplasia patients. Oral Dis..

[11] Kim BG (2006). Runx2 phosphorylation induced by fibroblast growth factor-2/protein kinase C pathways. Proteomics.

[12] Kim HJ (2003). The protein kinase C pathway plays a central role in the fibroblast growth factor-stimulated expression and transactivation activity of Runx2. J. Biol. Chem..

[13] Kim HJ, Rice DP, Kettunen PJ, Thesleff I (1998). FGF-, BMP- and Shh-mediated signalling pathways in the regulation of cranial suture morphogenesis and calvarial bone development. Development.

[14] Lee MH, Kwon TG, Park HS, Wozney JM, Ryoo HM (2003). BMP-2-induced Osterix expression is mediated by Dlx5 but is independent of Runx2. Biochem. Biophys. Res. Commun..

[15] Choi KY (2005). Runx2 regulates FGF2-induced Bmp2 expression during cranial bone development. Dev. Dyn..

[16] Wang XP (2005). Runx2 (Cbfa1) inhibits Shh signaling in the lower but not upper molars of mouse embryos and prevents the budding of putative successional teeth. J. Dent. Res..

[17] Canalis E, Centrella M, McCarthy T (1988). Effects of basic fibroblast growth factor on bone formation in vitro. J. Clin. Invest..

[18] Montero A (2000). Disruption of the fibroblast growth factor-2 gene results in decreased bone mass and bone formation. J. Clin. Invest..

[19] Selvamurugan N (2009). Identification and characterization of Runx2 phosphorylation sites involved in matrix metalloproteinase-13 promoter activation. FEBS Lett..

[20] Vimalraj S, Arumugam B, Miranda PJ, Selvamurugan N (2015). Runx2: structure, function, and phosphorylation in osteoblast differentiation. Int. J. Biol. Macromol..

[21] Park OJ, Kim HJ, Woo KM, Baek JH, Ryoo HM (2010). FGF2-activated ERK mitogen-activated protein kinase enhances Runx2 acetylation and stabilization. J. Biol. Chem..

[22] Jeon EJ (2006). Bone morphogenetic protein-2 stimulates Runx2 acetylation. J. Biol. Chem..

[23] Jun JH (2010). BMP2-activated Erk/MAP kinase stabilizes Runx2 by increasing p300 levels and histone acetyltransferase activity. J. Biol. Chem..

[24] Yoon WJ (2014). Prolyl isomerase Pin1-mediated conformational change and subnuclear focal accumulation of Runx2 are crucial for fibroblast growth factor 2 (FGF2)-induced osteoblast differentiation. J. Biol. Chem..

[25] Xu YX, Manley JL (2007). Pin1 modulates RNA polymerase II activity during the transcription cycle. Genes Dev..

[26] Islam R, Yoon WJ, Ryoo HM (2017). Pin1, the master orchestrator of bone cell differentiation. J. Cell. Physiol..

[27] Lee SH (2013). Prolyl isomerase Pin1 enhances osteoblast differentiation through Runx2 regulation. FEBS Lett..

[28] Kim HJ, Kim WJ, Ryoo HM (2020). Post-translational regulations of transcriptional activity of RUNX2. Mol. Cells.

[29] Shin HR (2018). PIN1 is a new therapeutic target of craniosynostosis. Hum. Mol. Genet..

[30] Kim B (2020). PIN1 attenuation improves midface hypoplasia in a mouse model of Apert syndrome. J. Dent. Res..

[31] Johnson D, Wilkie AO (2011). Craniosynostosis. Eur. J. Hum. Genet..

[32] Wilkie AO (2010). Prevalence and complications of single-gene and chromosomal disorders in craniosynostosis. Pediatrics.

[33] Forte AJ (2014). Analysis of midface retrusion in Crouzon and Apert syndromes. Plast. Reconstr. Surg..

[34] Grova M (2012). Models of cranial suture biology. J. Craniofac. Surg..

[35] Kim HJ (2003). Erk pathway and activator protein 1 play crucial roles in FGF2-stimulated premature cranial suture closure. Dev. Dyn..

[36] Shukla V, Coumoul X, Wang RH, Kim HS, Deng CX (2007). RNA interference and inhibition of MEK-ERK signaling prevent abnormal skeletal phenotypes in a mouse model of craniosynostosis. Nat. Genet..

[37] Yoon WJ (2013). Pin1-mediated Runx2 modification is critical for skeletal development. J. Cell. Physiol..

[38] Morita J (2014). Soluble form of FGFR2 with S252W partially prevents craniosynostosis of the apert mouse model. Dev. Dyn..

[39] Mooney MP (2007). Anti-TGF-beta2 antibody therapy inhibits postoperative resynostosis in craniosynostotic rabbits. Plast. Reconstr. Surg..

[40] Melville H, Wang Y, Taub PJ, Jabs EW (2010). Genetic basis of potential therapeutic strategies for craniosynostosis. Am. J. Med. Genet. A.

[41] Eswarakumar VP (2006). Attenuation of signaling pathways stimulated by pathologically activated FGF-receptor 2 mutants prevents craniosynostosis. Proc. Natl Acad. Sci. USA.

[42] Perlyn CA, Morriss-Kay G, Darvann T, Tenenbaum M, Ornitz DM (2006). A model for the pharmacological treatment of crouzon syndrome. Neurosurgery.

[43] Balek L (2017). ARQ 087 inhibits FGFR signaling and rescues aberrant cell proliferation and differentiation in experimental models of craniosynostoses and chondrodysplasias caused by activating mutations in FGFR1, FGFR2 and FGFR3. Bone.

[44] Yin L (2008). A Pro253Arg mutation in fibroblast growth factor receptor 2 (Fgfr2) causes skeleton malformation mimicking human Apert syndrome by affecting both chondrogenesis and osteogenesis. Bone.

[45] Wang Y (2012). p38 Inhibition ameliorates skin and skull abnormalities in Fgfr2 Beare–Stevenson mice. J. Clin. Invest..

[46] Chao SH, Greenleaf AL, Price DH (2001). Juglone, an inhibitor of the peptidyl-prolyl isomerase Pin1, also directly blocks transcription. Nucleic Acids Res..

[47] Tatara Y, Lin YC, Bamba Y, Mori T, Uchida T (2009). Dipentamethylene thiuram monosulfide is a novel inhibitor of Pin1. Biochem. Biophys. Res. Commun..

[48] Sun Y, Jiang X, Chen S, Price BD (2006). Inhibition of histone acetyltransferase activity by anacardic acid sensitizes tumor cells to ionizing radiation. FEBS Lett..

[49] Dinca A, Chien WM, Chin MT (2016). Intracellular delivery of proteins with cell-penetrating peptides for therapeutic uses in human disease. Int. J. Mol. Sci..

[50] Darby JF (2014). Discovery of selective small-molecule activators of a bacterial glycoside hydrolase. Angew. Chem. Int. Ed. Engl..

[51] Krappmann D, Scheidereit C (2005). A pervasive role of ubiquitin conjugation in activation and termination of IkappaB kinase pathways. EMBO Rep..

[52] Lee ZH, Kim HJ, Ryoo HM (2015). A novel osteogenic activity of suberoylanilide hydroxamic acid is synergized by BMP-2. J. Bone Metab..

[53] Huynh NC, Everts V, Ampornaramveth RS (2017). Histone deacetylases and their roles in mineralized tissue regeneration. Bone Rep..

[54] Serin HM, Koc ZP, Temelli B, Esen I (2015). The bone mineral content alterations in pediatric patients medicated with levetiracetam, valproic acid, and carbamazepine. Epilepsy Behav..

[55] Fan X, Li L, Ye Z, Zhou Y, Tan WS (2018). Regulation of osteogenesis of human amniotic mesenchymal stem cells by sodium butyrate. Cell Biol. Int..

[56] Kim HN (2011). Histone deacetylase inhibitor MS-275 stimulates bone formation in part by enhancing Dhx36-mediated TNAP transcription. J. Bone Min. Res..

[57] Bae HS (2017). An HDAC inhibitor, entinostat/MS-275, partially prevents delayed cranial suture closure in heterozygous Runx2 null mice. J. Bone Min. Res..

[58] Kumar R, Madewell JE, Swischuk LE, Lindell MM, David R (1989). The clavicle: normal and abnormal. Radiographics.

[59] Dokmanovic M, Clarke C, Marks PA (2007). Histone deacetylase inhibitors: overview and perspectives. Mol. Cancer Res..

[60] Gu Z, Biswas A, Zhao M, Tang Y (2011). Tailoring nanocarriers for intracellular protein delivery. Chem. Soc. Rev..

[61] Zhang Y, Roise JJ, Lee K, Li J, Murthy N (2018). Recent developments in intracellular protein delivery. Curr. Opin. Biotechnol..

[62] Kim WJ (2017). Fibroin particle-supported cationic lipid layers for highly efficient intracellular protein delivery. Biomaterials.

[63] Kim WJ (2017). Direct delivery of recombinant Pin1 protein rescued osteoblast differentiation of Pin1-deficient cells. J. Cell. Physiol..

[64] Khalid O (2008). Modulation of Runx2 activity by estrogen receptor-alpha: implications for osteoporosis and breast cancer. Endocrinology.

[65] Kawahata H (2003). Enhanced expression of Runx2/PEBP2alphaA/CBFA1/AML3 during fracture healing. J. Orthop. Sci..

[66] McGee-Lawrence ME (2014). Runx2 is required for early stages of endochondral bone formation but delays final stages of bone repair in Axin2-deficient mice. Bone.

[67] Bao L (2004). Prevalent overexpression of prolyl isomerase Pin1 in human cancers. Am. J. Pathol..

[68] Lu KP, Zhou XZ (2007). The prolyl isomerase PIN1: a pivotal new twist in phosphorylation signalling and disease. Nat. Rev. Mol. Cell Biol..

[69] Paneni F (2015). Targeting prolyl-isomerase Pin1 prevents mitochondrial oxidative stress and vascular dysfunction: insights in patients with diabetes. Eur. Heart J..

[70] Nechama M (2018). The IL-33–PIN1–IRAK-M axis is critical for type 2 immunity in IL-33-induced allergic airway inflammation. Nat. Commun..

[71] Manganaro L (2010). Concerted action of cellular JNK and Pin1 restricts HIV-1 genome integration to activated CD4+ T lymphocytes. Nat. Med..

